# Newly acquired N-terminal extension targets threonyl-tRNA synthetase-like protein into the multiple tRNA synthetase complex

**DOI:** 10.1093/nar/gkz588

**Published:** 2019-07-09

**Authors:** Xiao-Long Zhou, Yun Chen, Qi-Yu Zeng, Zhi-Rong Ruan, Pengfei Fang, En-Duo Wang

**Affiliations:** 1 State Key Laboratory of Molecular Biology, CAS Center for Excellence in Molecular Cell Science, Shanghai Institute of Biochemistry and Cell Biology, Chinese Academy of Sciences, University of Chinese Academy of Sciences, 320 Yue Yang Road, Shanghai 200031, China; 2 State Key Laboratory of Bioorganic and Natural Products Chemistry, Center for Excellence in Molecular Synthesis, Shanghai Institute of Organic Chemistry, Chinese Academy of Sciences, 345 Lingling Road, Shanghai 200032, China; 3 School of Life Science and Technology, ShanghaiTech University, 100 Haike Road, Shanghai 201210, China

## Abstract

A typical feature of eukaryotic aminoacyl-tRNA synthetases (aaRSs) is the evolutionary gain of domains at either the N- or C-terminus, which frequently mediating protein–protein interaction. *TARSL2* (mouse *Tarsl2*), encoding a threonyl-tRNA synthetase-like protein (ThrRS-L), is a recently identified aaRS-duplicated gene in higher eukaryotes, with canonical functions *in vitro*, which exhibits a different N-terminal extension (N-extension) from *TARS* (encoding ThrRS). We found the first half of the N-extension of human ThrRS-L (hThrRS-L) is homologous to that of human arginyl-tRNA synthetase. Using the N-extension as a probe in a yeast two-hybrid screening, AIMP1/p43 was identified as an interactor with hThrRS-L. We showed that ThrRS-L is a novel component of the mammalian multiple tRNA synthetase complex (MSC), and is reliant on two leucine zippers in the N-extension for MSC-incorporation in humans, and mouse cell lines and muscle tissue. The N-extension was sufficient to target a foreign protein into the MSC. The results from a *Tarsl2*-deleted cell line showed that it does not mediate MSC integrity. The effect of phosphorylation at various sites of hThrRS-L on its MSC-targeting is also explored. In summary, we revealed that ThrRS-L is a *bona fide* component of the MSC, which is mediated by a newly evolved N-extension domain.

## INTRODUCTION

The genetic code is a universal algorithm for linking nucleotide triplets in mRNAs to the amino acids in proteins via mRNA translation (protein synthesis). Aminoacyl-tRNA synthetases (aaRSs) comprise a ubiquitously expressed family of enzymes that catalyze the esterification reaction to ligate transfer RNAs (tRNAs) with their cognate amino acids to generate aminoacyl-tRNAs for protein biosynthesis at the ribosome (1,2). Usually, tRNA charging (aminoacylation) occurs in two-step reactions involving amino acid activation, with the generation of intermediate aminoacyl-AMP, and transfer of the aminoacyl moiety of the intermediate to A76 of tRNA (1,3). Some aaRSs also catalyze a proofreading and editing reaction to remove misactivated amino acids and mischarged tRNAs caused by an inability to correctly discriminate cognate amino acids from noncognate ones, which ensures a high level of accuracy in protein biosynthesis (4,5). The activities of aminoacylation and editing are canonical functions of aaRSs that maintain the speed and fidelity of genetic code transduction.

AaRSs have been present in the last common ancestor of the tree of life. Modern aaRSs are split into two distinct classes based on the architectures of the two distinct active sites, which are considered to be the scaffold of ancient aaRSs (6,7). All present-day aaRSs contain catalytic and anticodon binding domains to perform aminoacylation, together with editing domains in some aaRSs to carry out the editing activity. However, during evolution of aaRSs from prokaryotes to archaea and eukaryotes, certain aaRSs obtain new appended domains, usually at the N- or C-terminus (8), with unique structural characteristics that are not a part of the catalytic core, but frequently mediate protein–protein interactions in various functions unrelated to canonical aminoacylation and editing, such as translation and transcription regulation, angiogenesis, inflammation and tumorigenesis (9–13).

Another well-characterized feature of aaRSs, especially in eukaryotes, is the presence of the high order multiprotein complex. For instance, in *Saccharomyces cerevisiae*, glutamyl-tRNA synthetase (GluRS) and methionyl-tRNA synthetase (MetRS) stably interact with a nonsynthetase accessory protein, Arc1p (14). An aaRS-containing complex is also observed in archaea (15). In human cytoplasm, nine aaRSs [including arginyl-, aspartyl-, glutaminyl-, glutamyl-prolyl-, isoleucyl-, leucyl-, methionyl- and lysyl-tRNA synthetases (ArgRS, AspRS, GlnRS, GluProRS, IleRS, LeuRS, MetRS, LysRS, respectively)] with three auxiliary scaffold protein co-factors [aminoacyl-tRNA synthetase complex-interacting multifunctional protein (AIMP)1/p43, AMIP2/p38 and AIMP3/p18] (hereinafter referred to as p43, p38 and p18) form a multiple tRNA synthetase complex (MSC) (16). It has been proposed that the newly acquired domains are related with the occurrence of the MSC (8). The interaction pattern between the extra domains in human cytoplasmic aaRSs has been studied or confirmed by cross-linking (17), yeast two-hybrid (18), co-immunoprecipitation (19) assays and co-crystal structure determination with different components (20). The detailed mechanism of the formation of the MSC in higher eukaryotes is unclear; however, some models, such as facilitating tRNA channeling between various translation apparatus or functioning as a reservoir for regulatory proteins that, upon inducible release, acquire new auxiliary functions, or facilitating the tRNA transport from nucleus to cytoplasm, have been proposed (21,22). Indeed, several components of the human MSC, such as LysRS, GluProRS, LeuRS, GlnRS and MetRS, can be either released from the complex or have non-canonical functions in other cellular compartments under specific stresses or stimulations (9,10,23–25).

It is believed that there are 37 aaRS genes in human cells, encoding full sets of aaRSs for both cytoplasmic and mitochondrial protein synthesis (26). However, an aaRS-duplicated gene, *TARSL2* was identified in 2013 for the first time in our laboratory, which encodes a threonyl-tRNA synthetase (ThrRS)-like protein (ThrRS-L) (27). ThrRS-L shares high similarity with canonical ThrRS in the N1, N2, aminoacylation and C-terminal tRNA binding domains; however, it has evolved a quite different N-terminal extension (N-extension) from that of ThrRS. Later, human ThrRS-L (hThrRS-L) was found to be a component of the MSC by affinity purification-mass spectrometry in human cells, despite the fact that quantitative determination showed an obviously low abundance of ThrRS-L in human cell lines (28,29). In mouse, *Tarsl2* encodes mouse ThrRS-L (mThrRS-L), which has 86% identity with hThrRS-L. Recently, we further revealed that the mRNA of mouse *Tarsl2* is present at significantly lower levels than that of *Tars* in various mouse tissues and mouse cell lines with most enriched proteins in the muscle and heart (30). Interestingly, mThrRS-L is able to locate to the nucleus and, *in vitro*, can catalyze aminoacylation with similar efficiency despite having decreased editing activity and a distinct cross-species tRNA recognition capability compared with those of mouse ThrRS (mThrRS) (30). However, whether mThrRS-L is responsible for canonical tRNA aminoacylation to generate Thr-tRNA^Thr^ for protein biosynthesis *in vivo* is unclear. Furthermore, its presence only in higher eukaryotes implies it has a non-canonical function. Harbouring a unique N-extension, whether it is a *bona fide* component of the MSC, and what is the mechanism of ThrRS-L-incorporation into MSC, are also unknown.

In the present study, based on the unique N-extension of hThrRS-L, we first analyzed its primary sequence and then used it as a bait protein to perform a yeast two-hybrid screening. We identified p43 is one of the proteins that interact with hThrRS-L, and further provided extensive data showing that ThrRS-L is a *bona fide* member of the MSC in humans and mice. We also revealed detailed mechanism of hThrRS-L targeting into the MSC. Furthermore, based on a *Tarsl2*-deleted cell line, we showed that absence of mThrRS-L has no effect on MSC integrity.

## MATERIALS AND METHODS

### Materials

Dithiothreitol (DTT), Tris-base, phenyl methylsulfonyl fluoride (PMSF) and NaCl, were purchased from Sangon (Shanghai, China). KOD-plus mutagenesis kits were obtained from TOYOBO (Osaka, Japan). All restriction endonucleases, T4 DNA ligase, T4 Polynucleotide Kinase, Dynabeads protein G and Lipofectamine 2000 transfection reagent were obtained from Thermo Scientific (Waltham, MA, USA). All materials for the yeast two-hybrid screening and assay, including the Matchmaker Gold Yeast Two-Hybrid System, media, reagents, and a normalized Mate & Plate human cDNA library, were purchased from Clontech (Shiga, Japan). *Escherichia coli* Rosetta (DE3) cells were purchased from Stratagene (Santa Clara, CA, USA). Polyvinylidene fluoride (PVDF) membranes were obtained from Millipore (Darmstadt, Germany). DNA sequencing was performed by Biosune (Shanghai, China).

### Antibodies

Anti-mThrRS-L, anti-FLAG, anti-His_6_-tag, anti-α-tubulin and anti-glycyl-tRNA synthetase (GlyRS) were detailed in a previous report (30). A glutathione-*S*-transferase (GST) fusion protein containing the N-terminal region of hThrRS-L (Met^1^–Leu^50^) or that of human ThrRS (hThrRS) (Met^1^–Glu^52^) was used as an antigen to generate anti-hThrRS-L or anti-hThrRS antibodies. Anti-Myc (M4439), anti-β-actin (A1978), anti-enhanced green fluorescent protein (EGFP) (G1544), HRP (horse radish peroxidase)-labeled anti-mouse and anti-rabbit secondary antibodies were obtained from Sigma-Aldrich (St. Louis, MO, USA). Details for the anti-p43, anti-LysRS, anti-ArgRS, anti-GluProRS, anti-LeuRS and anti-GlnRS were provided in a previous report (31).

### Cloning

Genes encoding hThrRS and hThrRS-L were amplified from cDNA, obtained by reverse-transcription PCR from total RNA extracted from Human Embryonic Kidney 293T (HEK293T) cells. DNA encoding a C-terminal FLAG- or His_6_-tagged hThrRS-L was cloned into pCMV-3Tag-3A. DNA encoding an N-terminal HA- or Myc-tagged hThrRS was inserted into pcDNA3.1. The DNA fragment (named as *TLN161*) encoding the N-terminal appended domain of hThrRS-L (Met^1^–Ile^161^, TLN161) was inserted into the gap between the NdeI and BamHI sites of pGBKT7 to produce the recombinant plasmid pGBKT7-*TLN161*. Co-expression of the two genes encoding hThrRS-L and any of the three (p43, LysRS or ArgRS, respectively) was performed by inserting the two genes into the gaps between the two cloning sites of pRSFDuet1; and the recombinant plasmid was then transformed into *E. coli*. Gene mutagenesis or fragment deletion was performed according to the protocol provided with the KOD-plus mutagenesis kit.

### Yeast two-hybrid assay

All the yeast two-hybrid screening or assays were performed according to the user manual provided with Matchmaker Gold Yeast Two-Hybrid System. Briefly, pGBKT7-*TLN161* was transformed into the Y2HGold yeast strain to express a Gal4 DNA binding domain fused TLN161, which was then tested for autoactivation and toxicity. Y2HGold cells expressing *TLN161* were mated with Y187 yeast strains containing a normalized Mate & Plate human cDNA library and plated on Quadruple Dropout (QDO) (SD/Ade^−^/His^−^/Leu^-^/Trp^−^) medium supplemented Aureobasidin A and X-α-Gal (QDO/X/A). The blue colonies that grew on the QDO/X/A plate were selected and their plasmids were extracted. The DNA fragment in the pGADT7 vector was sequenced. To confirm the interaction between TLN161 with other targeted proteins, the gene encoding targeted protein was cloned into the pGADT7. Subsequently, the construct was transformed into the Y2HGold yeast strain together with pGBKT7-*TLN161*. The transformant was plated on QDO/X/A and its growth was observed.

### Cell culture and transfection

HEK293T cells were cultured in Dulbecco's modified Eagle's medium (DMEM, high glucose) supplemented with 10% fetal bovine serum (FBS) in a 37°C incubator with 5% CO_2_. Transfection was performed using the Lipofectamine 2000 transfection according to the manufacturer's protocol. Twenty-four hours after transfection, the cells were washed with 5 ml of ice-cold phosphate-buffered saline (PBS) twice, and lyzed with 1 ml of ice-cold lysis buffer [50 mM Tris–HCl (pH 7.5), 150 mM NaCl, 5 mM EDTA, and 1% Triton X-100] supplemented with a protease inhibitor cocktail. The supernatant was collected using centrifugation at 12 000 × *g* for 30 min. All procedures were performed on ice.

### Gel filtration chromatography of cell lysates

Cytosolic or mouse tissue extracts were applied to a Superose-6 column for high-performance liquid chromatography (HPLC) and eluted at a flow rate of 0.5 ml/min by using a buffer containing 50 mM Tris–HCl (pH 7.5), 50 mM NaCl, 1 mM phenyl methylsulfonyl fluoride (PMSF) and 1 mM dithiothreitol (DTT). Fractions were collected for immunoblotting (western blotting).

### Western blotting

The different proteins of interest in whole cell lysates were separated using 10% SDS-PAGE with pre-stained molecular protein standards; and then transferred to a PVDF membrane. The PVDF membrane was then cropped to blot different proteins in the same lane. After blocking with 5% (w/v) non-fat dried milk, the membranes with targeted proteins were incubated and detected with the corresponding primary antibodies overnight at 4°C. The membranes were then washed three times using PBS plus 0.05% Tween-20 (PBST) (137 mM NaCl, 2.7 mM KCl, 10 mM Na2HPO4, 2 mM KH2PO4 and 0.5‰ Tween-20) and incubated with HRP-conjugated secondary antibody at room temperature for 30 min. After washing three times with PBST, the membranes were treated with the chemiluminescent substrate, and imaging was performed using the Amersham imager 680 system (GE, CA, USA).

### Immunoprecipitation

Whole cell lysates with different proteins of interest were incubated with specific primary antibody with agitation overnight, and then the mixture was incubated with Dynabeads protein G for 3 h. Recovered immune complexes were washed three times using PBST. All procedures are performed at 4°C. Proteins were eluted from the beads in 2× protein loading buffer (100 mM Tris–HCl, 4% SDS, 0.2% bromophenol blue, 20% glycerol, 200 mM DTT), boiled for 10 min at 95°C. The retrieved proteins were subjected to SDS-PAGE and further analyzed by western blotting or visualized and identified by silver staining and mass spectrometry (MS), respectively.

### Obtaining a *Tarsl2* deletion NIH/3T3 cell line

The *Tarsl2* deletion NIH/3T3 cell line was constructed using CRISPR/Cas9 mediated gene targeting technology (32). Briefly, a guide RNA (sgRNA) (5′-GGCGGAGCAGCGCCGCACCGAGG-3′ with the PAM sequences underlined) was designed that targeted exon 1 of the transcript of *Tarsl2* (ENSMUST00000032728.8). Sense and antisense oligonucleotides for the guide RNA were cloned into pX330-*mCherry* plasmid to produce pX330-*mCherry*-sgRNA. The NIH/3T3 cells were transfected with pX330-*mCherry*-sgRNA using lipofectamine 2000 according to the manufacturer's instructions. Twelve hours after transfection, the NIH/3T3 cells expressing sgRNA with the red fluorescence protein were sorted using flow cytometry (FACS Aria SORP) and plated at 96-well cell culture plates. The genotypes of NIH/3T3 cell lines were analyzed by DNA sequencing of PCR products amplified from targeted sites.

## RESULTS

### The N-terminal extension of hThrRS-L is homologous to that of the long isoform of human cytoplasmic ArgRS

**Figure 1.**
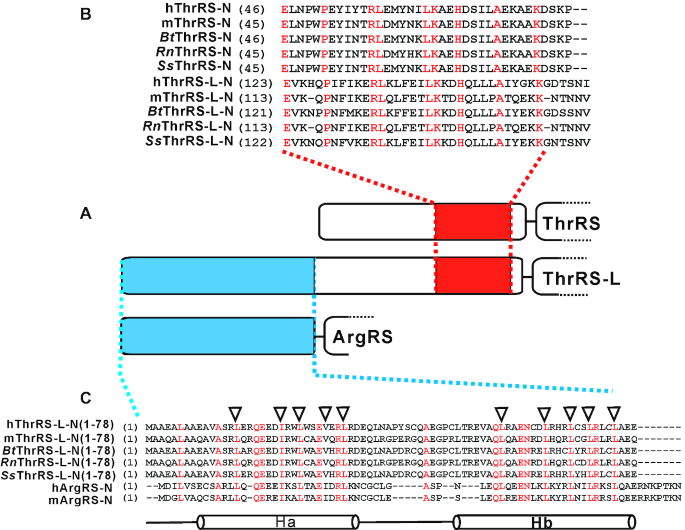
Primary sequence alignment and comparison of the N-extension of ThrRS-Ls with that of ThrRSs and ArgRSs. (**A**) Schema showing the N-extension of ThrRS-L, ThrRS, and ArgRS. (**B**) Sequence comparison between N-extensions of ThrRS-L (ThrRS-L-N) and ThrRS (ThrRS-N). (**C**) Sequence comparison between N-extensions of ThrRS-L and ArgRS. The two helixes (Ha and Hb) as revealed by the structure of ArgRS-GlnRS-p43 (PDB 4R3Z) are shown at the bottom. hThrRS and hThrRS-L represent human ThrRS and ThrRS-L; similarly, mThrRS and mThrRS-L represent mouse ThrRS and ThrRS-L. The amino acid residues for mutagenesis in hThrRS-L-Ha/A or hThrRS-L-Hb/A were indicated by the inverted triangles. *Bt, Bos Taurus*; Rn, *Rattus norvegicus*; *Ss, Sus scrofa*.

Sequence analysis among bacterial ThrRSs, canonical eukaryotic cytoplasmic ThrRSs and mammalian ThrRS-Ls showed that both eukaryotic cytoplasmic ThrRSs and ThrRS-Ls have acquired newly evolved N-terminal extensions (N-extensions) when compared with bacterial ThrRSs (except truncated yeast mitochondrial or *Mycoplasma* ThrRSs) (33,34) (Figure 1A). For the human enzymes, the N-extensions of hThrRS (Uniprot: P26639) and hThrRS-L (Uniprot: A2RTX5) are peptides with 82 or 161 amino acid residues. The two extensions share conserved residues in the last sections but with 64% identity in the full-length enzymes. Besides, the sequence identity between hThrRS-L and human mitochondrial ThrRS precursor is only 46%. A similar observation was made for the N-extensions of mThrRS (Uniprot: Q9D0R2) and mThrRS-L (Uniprot: Q8BLY2), with 81 and 149 residues, respectively. However, the N-extensions between various ThrRS-Ls or ThrRSs are highly conserved (Figure 1B). These observations suggested that the N-extensions of ThrRSs and ThrRS-Ls are likely to be of different origins.

We searched for any potential homologous peptide of N-extensions of ThrRS-Ls using BLASTP analysis. The N-terminal region (Met^1^–Glu^78^) of hThrRS-L was significantly homologous to the N-extension of the long isoform of human cytoplasmic arginyl-tRNA synthetase (ArgRS, Uniprot: P54136), encoded by *RARS*, whose mRNA generates two isoforms of ArgRS by alternative translation initiation (Figure 1C) (35). The sequence identity between full-length hThrRS-L and human ArgRS is only 13% (35). Based on the co-crystal structure (PDB 4R3Z) of ArgRS, p43 and GlnRS (Uniprot: P47897), its N-extension forms two α-helices (Ha and Hb), with one leucine-zipper in each helix, which mediate extensive protein–protein interaction with both p43 and GlnRS. In particular, residues in the two leucine-zippers are conserved between hThrRS-L and ArgRS (Figure 1C), suggesting the counterpart in the N-terminal portion of TLN161 (Met^1^–Glu^78^) also likely forms two similar α-helices (also designated Ha and Hb) and has the potential to mediate protein–protein interaction with other unidentified proteins.

### Overexpressed hThrRS-L forms homodimers and heterodimers with overexpressed hThrRS *in vivo*

**Figure 2.**
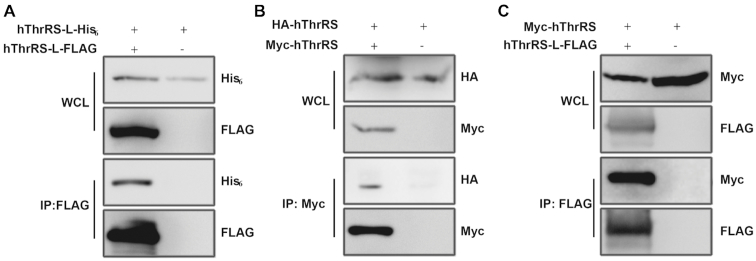
Overexpressed hThrRS-L forms homodimers or heterodimers with overexpressed hThrRS *in vivo*. (**A**) After co-expression of genes encoding hThrRS-L-His_6_ and hThrRS-L-FLAG in HEK293T cells, hThrRS-L-His_6_ was readily pulled down by hThrRS-L-FLAG in a Co-IP assay. (**B**) After co-expression of genes encoding HA-hThrRS and Myc-hThrRS in HEK293T cells, HA-hThrRS was readily pulled down by Myc-hThrRS in a Co-IP assay. (**C**) After co-expression of genes encoding Myc-hThrRS and hThrRS-L-FLAG in HEK293T cells, Myc-hThrRS was readily precipitated by hThrRS-L-FLAG in a Co-IP assay.

Most class II synthetases, including ThrRS, are homodimers (6). The elements for homodimerization of ThrRS are found in the class-defining motif 1, and two short adjacent strands. Our previous study of yeast cytoplasmic ThrRS (*Sc*ThrRS) demonstrated that homodimerization of ThrRS is a prerequisite for catalysis (36). Based on the conservation of the main body of ThrRS-L and ThrRS, we simultaneously overexpressed C-terminal FLAG- and His_6_-tagged hThrRS-Ls (hThrRS-L-FLAG and hThrRS-L-His_6_) in HEK293T cells. The results of a co-immunoprecipitation (Co-IP) assay showed that hThrRS-L dimerized *in vivo* (Figure 2A). As expected, N-terminal HA-tagged hThrRS (HA-hThrRS) formed a homodimer with N-terminal Myc-tagged hThrRS (Myc-hThrRS) (Figure 2B). When hThrRS-L-FLAG and Myc-hThrRS were simultaneously expressed in HEK293T cells, Myc-hThrRS could be readily precipitated by hThrRS-L-FLAG, demonstrating the formation of an hThrRS-L/hThrRS complex (Figure 2C). In fact, we also revealed that overexpressed hThrRS-L forms a complex with native hThrRS *in vivo* and that native mThrRS-L and mThrRS formed a complex in muscle tissue (see text below). Consistently, our recent data showed that native hThrRS-L and hThrRS could be pulled down by overexpressed mThrRS-L (30). Taken together, these data clearly showed that ThrRS-L has the potential to form a homodimer and a complex with ThrRS. Considering the dimerization characteristics of class II tRNA synthetase, we hypothesized that the complex of ThrRS-L and ThrRS is a heterodimer. We further showed that the N-extension of hThrRS-L is not involved in interaction with hThrRS (see details below), consistent with dimerization of ThrRS relying on the aminoacylation domain.

### p43 was identified to interact with ThrRS-L based on a yeast two-hybrid screening

**Figure 3.**
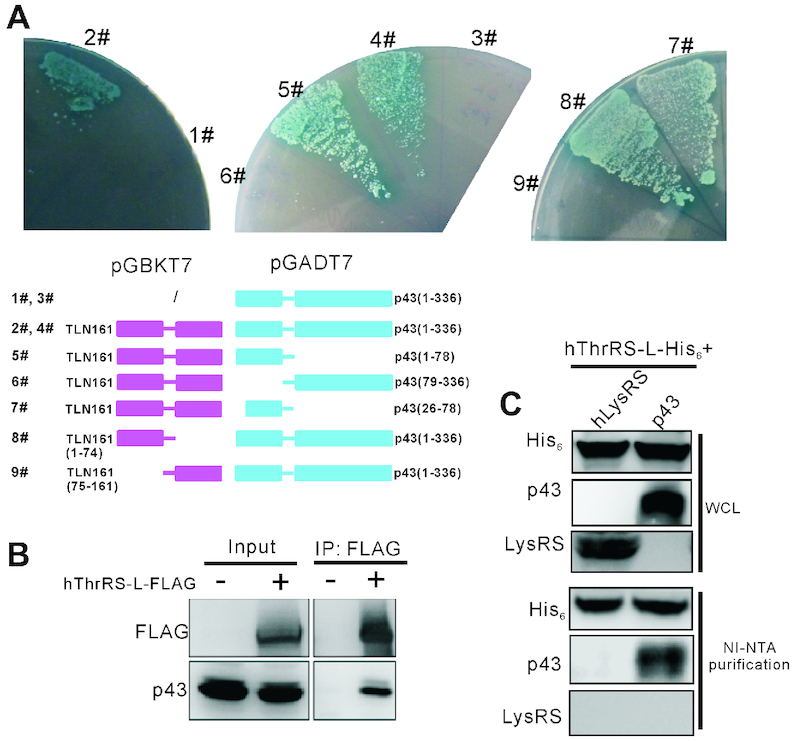
Identification of p43 as an interactor of hThrRS-L. (**A**) Left, Y2H Gold yeast cells expressing either native p43 (336-aa in length) alone (1# and 3#) or p43 plus TLN161 (2# and 4#) were grown on SD/Ade^−^/His^−^/Leu^−^/Trp^−^/X-α-Gal (SD/QDO/X) plates, identifying p43 as a binding protein of TLN161; middle and right, interaction-domain mapping of p43 (5#-7#) and TLN161 (8# and 9#) on an SD/QDO/X plate as constructs expressing various proteins or domains shown at the bottom. (**B**) Co-IP analysis confirming the interaction between native p43 and overexpressed hThrRS-L-FLAG in HEK293T cells. (**C**) Ni-NTA chromatography purification analysis confirming interaction between overexpressed p43 and overexpressed hThrRS-L-His_6_ in *E. coli*. As a control, LysRS and hThrRS-L-His_6_ were also overexpressed in *E. coli* and LysRS was not detected together with hThrRS-L-His_6_.

To identify any potential proteins that interact with hThrRS-L, and to exclude those interacting with hThrRS, we used hThrRS-L-specific TLN161 as a probe to perform a yeast two-hybrid screen on a human cDNA library. After confirming that TLN161 did not act as a transcription factor (data not shown), we found 78 positive clones containing proteins that interacted with TLN161. Among these clones, some encoded the same proteins or domains and some do not harbor in-frame coding sequences in comparison with human Open Reading Frames (ORFs). At the end, 12 proteins were screened. Among these proteins, p43 was the only protein functionally associated with tRNA synthetase (2# and 4# in Figure 3A). All other 11 proteins are non-aaRS proteins. Based on our previous study showing that ThrRS-L is active in canonical functions as an aaRS *in vitro* (30), we initially focused on potential interaction between ThrRS-L and p43. To confirm the interaction between hThrRS-L and p43 *in vivo*, we overexpressed hThrRS-L-FLAG in HEK293T cells and performed a Co-IP assay. The data showed that native p43 was indeed co-precipitated by the FLAG antibody (Figure 3B). We also expressed hThrRS-L-His_6_ and p43 in *E. coli*. Despite that hThrRS-L forms extensive inclusion bodies in *E. coli*; however, after purification using Nickel-nitrilotriacetic acid (Ni-NTA) affinity chromatography, western blotting analysis showed that p43 could be co-precipitated with hThrRS-L-His_6_; however human cytoplasmic LysRS could not. The above data clearly showed that hThrRS-L interacts with p43 *in vivo* (Figure 3C).

In spite of its main localization in the MSC, p43 is a multi-functional protein present in several cellular locations, including the nucleus, secretion out of the plasma membrane, and in mitochondria (37,38). The C-terminal region of p43, homologous to the endothelial monocyte-activating polypeptide-II (EMAP-II), is responsible for tRNA binding, while its N-terminal portion [p43(1–78), Met^1^–Lys^78^] mediates protein–protein interactions (37). Indeed, based on the co-crystal structure (PDB: 4R3Z) (20), the N-extension of ArgRS, comprising two α-helices, and the H1 helix of the core domain of ArgRS sandwich p43 by interacting with its long helix in the N-terminus. p43 has two isoforms with different N-terminus lengths, comprising 312 and 336 amino acid residues, respectively (Uniprot No. Q12904-1 and Q12904-2, respectively). The clone we screened contained the ORF of the 336-aa isoform; therefore, in this work we focused on the interaction of TLN161 in hThrRS-L with the longer p43. The homology of the N-extension between hThrRS-L and ArgRS (Figure 1C) prompted us to split p43 into two fragments, p43(1-78)(Met^1^–Lys^78^) and p43(79-336)(Leu^79^–Ly^336^), to map the region mediating its interaction with hThrRS-L. Expressing each fragment as a fusion protein with the GAL4 DNA activation domain, we found that p43(1-78) interacts with TLN161 (5# in Figure 3A); however p43(79–336) does not (6# in Figure 3A). The shorter p43 isoform only lacked the 25 N-terminal amino acid residues. The peptide consisting of Met^26^–Lys^78^ [p43(26–78)] still interacted with TLN161 (7# in Figure 3A). To determine whether the ArgRS-homologous region, the N-terminal part of TLN161 [TLN161(1–74)(Met^1^–Cys^74^)], or the C-terminal part of TLN161 [TLN161(75–161)(Leu^75^–Ile^161^)], was the binding element with p43, the two peptides were fused separately with the GAL4 DNA binding domain. The results showed that the N-terminal part of TLN161 is responsible for binding p43 (8# in Figure 3A), and the C-terminal part of TLN is not (9# in Figure 3A).

Taken together, these data showed that hThrRS-L and p43 interact *in vivo* and the N-terminuses of both proteins are responsible for the interaction.

### ThrRS-L is a *bona fide* component of multiple-synthetase complex (MSC)

**Figure 4.**
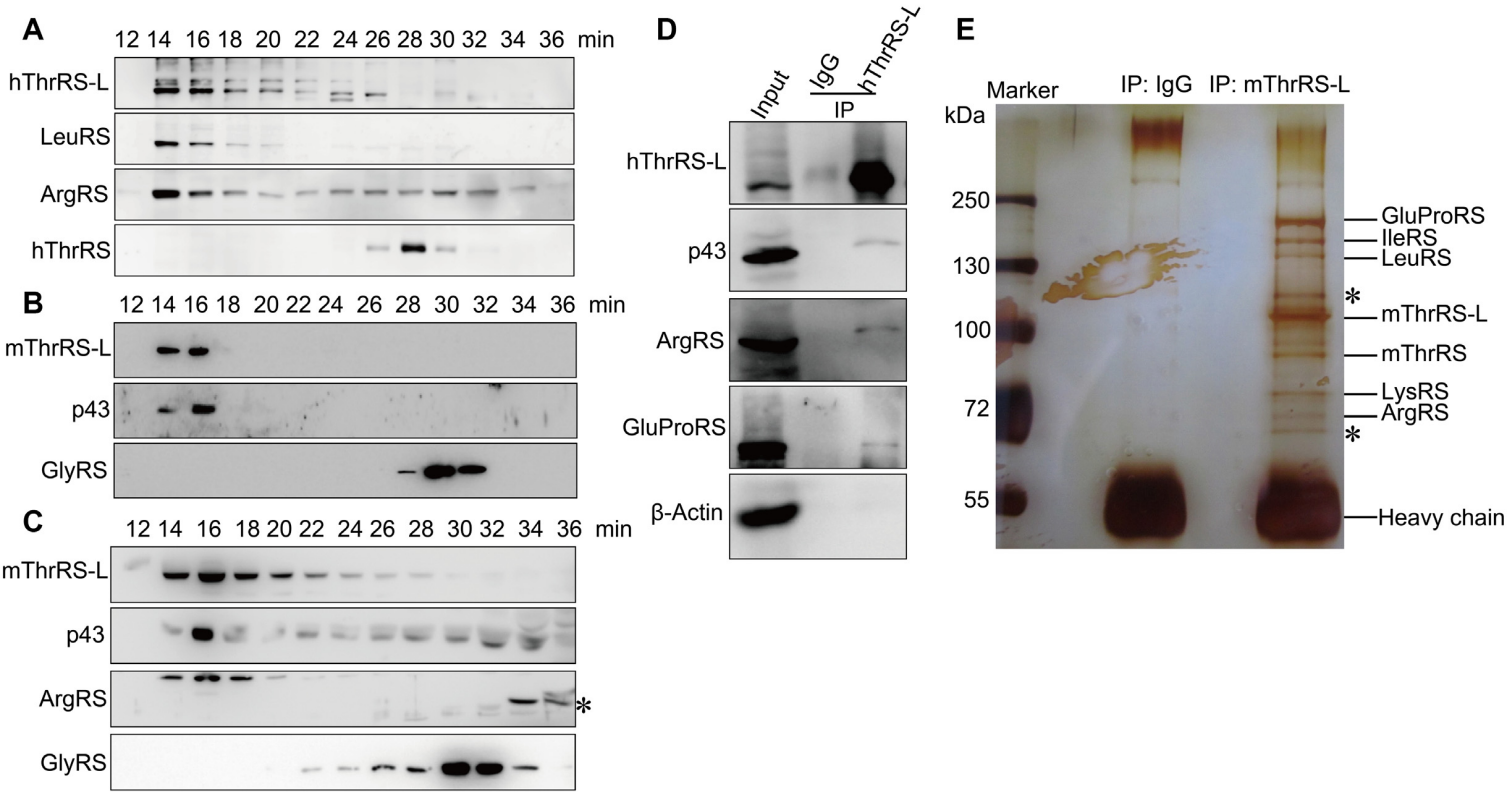
ThrRS-L is a component of the MSC. Whole cell lysate of HEK293T cells (**A**) or NIH/3T3 cells (**B**) or mouse muscle tissue (**C**) was fractionated by gel filtration chromatography and various components of the MSC or free tRNA synthetase (hThrRS or mouse GlyRS) were detected in the fractions. The anti-ArgRS antibody recognized both long and short (indicated by an asterisk) forms of mouse ArgRS in (C). (**D**) Co-IP was performed using an hThrRS-L antibody to pull down components of the MSC in HEK293T cells. β-actin was used as a negative (non-component) control. (**E**) Co-IP was performed using an anti-mouse (m)ThrRS-L antibody to pull down components of the MSC from mouse muscle tissue; after SDS-PAGE separation and silver-staining, bands present only in the mThrRS-L antibody-precipitated sample were cut out for identification by Mass Spectrometry. Mouse GluProRS, IleRS, LeuRS, LysRS, ArgRS and ThrRS were detected, in addition to two non-aaRS proteins, Atp2a1 (indicated by the upper asterisk) and Dlat (indicated by the lower asterisk).

Based on the interaction of hThrRS-L with p43, and the potential association of hThrRS-L with the MSC, we further explored whether hThrRS-L is a component of the MSC. Gel filtration chromatography was performed to enrich the MSC-containing fraction from the whole cell lysate of mammalian cells and mouse muscle tissue, in which *Tarsl2* (mThrRS-L gene) is abundantly expressed. ThrRS-L clearly eluted simultaneously with known MSC components from HEK293T cell extracts (LeuRS and ArgRS, Figure 4A), mouse NIH/3T3 cell extracts (p43, Figure 4B), and extracts of mouse muscle tissue (p43 and ArgRS, Figure 4C). These data implied that ThrRS-L was a potential member of the MSC. To confirm the direct interaction of ThrRS-L with the components of the MSC, a Co-IP assay was performed with native HEK293T cell lysates and anti-hThrRS-L antibodies; several MSC components, including GluProRS, ArgRS and p43, were co-precipitated (Figure 4D). To explore whether the native MSC could be enriched using anti-mThrRS-L antibodies (30) in mice, we performed a Co-IP assay with anti-mThrRS-L antibodies in an extract of mouse muscle tissue. After precipitation, the products were separated by SDS-PAGE and bands were cut out for MS identification. Mouse GluProRS, IleRS, LeuRS, LysRS and ArgRS were clearly detected as the strong bands (bands of lower intensity were not analyzed) (Figure 4E). Strikingly, native mThrRS was pulled down together with native mThrRS-L, suggesting the interaction between native ThrRS and native ThrRS-L *in vivo*, which was consistent with data after gene overexpression (Figure 2C). In addition, two novel non-aaRS proteins were also detected (indicated by asterisks in Figure 4E).

Both gel filtration and Co-IP analysis clearly showed that mammalian ThrRS-L is a *bona fide* component of the MSC in human and mouse cell lines or mouse muscle tissue.

### Two leucine-zipper motifs in TLN161 target hThrRS-L into the MSC

**Figure 5.**
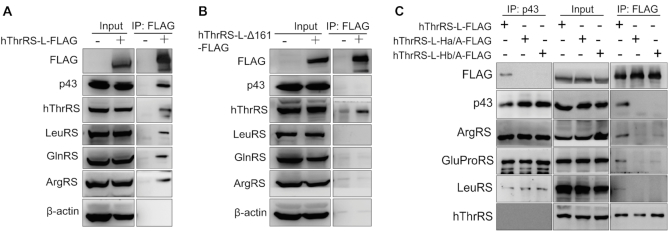
Two leucine-zippers in the N-extension mediate targeting of hThrRS-L into MSC. hThrRS-L-FLAG (**A**) or hThrRS-L-Δ161-FLAG (**B**) was overexpressed in HEK293T cells and Co-IP was performed using the anti-FLAG antibody. Various known MSC components and hThrRS were detected using their specific antibodies. β-Actin was used as a negative (non-component) control. (**C**) hThrRS-L-FLAG, hThrRS-L-Ha/A-FLAG, or hThrRS-L-Hb/A-FLAG were overexpressed in HEK293T cells and Co-IP was performed using either anti-p43 or anti-FLAG antibodies. Various known MSC components and hThrRS were detected using their specific antibodies.

We overexpressed the gene encoding hThrRS-L-FLAG in HEK293T cells. Co-IP assays showed that several MSC components, such as LeuRS, GlnRS, ArgRS, and p43, were readily pulled down using anti-FLAG antibodies (Figure 5A), which was consistent with data from gel filtration and Co-IP analyses using native cells (Figure 4A, B) or tissue (Figure 4C). Again, although it is not a member of the MSC, native hThrRS was also co-precipitated (Figure 5A), further confirming that ThrRS and ThrRS-L interact *in vivo* (Figures 2C, 4E).

The interaction of TLN161 with p43, one of the core components of the MSC, suggested that TLN161 is the most likely element that mediates MSC incorporation of ThrRS-L. To test this possibility, we overexpressed a deleted TLN161 mutant with a C-terminal FLAG-tag, hThrRS-L-Δ161-FLAG, in HEK293T cells. In HEK293T cells containing hThrRS-L-FLAG, LeuRS, GlnRS, ArgRS or p43 could be co-precipitated (Figure 5A) using anti-FLAG antibodies; however in HEK293T cells containing hThrRS-L-Δ161-FLAG, these bands disappeared (Figure 5B). Notably, hThrRS was pulled down with hThrRS-L-Δ161-FLAG (Figure 5B), indicating that the N-extension was not required for binding hThrRS, consistent with reports that the aminoacylation domain of ThrRS is the dimer interface (6,36). These data showed that the N-extension of hThrRS-L is necessary for its incorporation into the MSC but is not responsible for the potential interaction with hThrRS.

Recently, two leucine-zipper motifs in two α-helices (Ha and Hb) in the N-extension of ArgRS were shown to mediate protein–protein interactions in the MSC (20). Based on the two conserved leucine-zipper motifs between ArgRS and hThrRS-L (Figure 1C) and the region (Met^1^–Cys^74^) covering these leucine-zipper motifs being the p43-interaction element (Figure 3A), we mutated counterparts of the leucine-zipper motif in Ha (including Leu^15^, Ile^22^, Leu^25^, Val^29^ and Leu^32^) or in Hb (including Leu^57^, Leu^64^, Leu^68^, Leu^71^ and Leu^75^) in hThrRS-L to Ala residues, obtaining the mutants hThrRS-L-Ha/A and hThrRS-L-Hb/A, respectively (Figure 1C). hThrRS-L-FLAG, hThrRS-L-Ha/A-FLAG or -Hb/A-FLAG were overexpressed in HEK293T cells. Similar protein levels were observed in the wild-type and the two mutants of hThrRS-L, suggesting the two leucine zippers had little effect on the stability of hThrRS-L in the cells (Figure 5C). However, Co-IP with anti-p43 antibodies could only precipitate hThrRS-L-FLAG, but not hThrRS-L-Ha/A-FLAG or hThrRS-L-Hb/A-FLAG (Figure 5C), indicating the importance of both leucine-zippers for MSC incorporation. Consistently, components of the MSC (including p43, ArgRS, GluProRS and LeuRS) were only pulled down with anti-FLAG antibodies in cells expressing hThrRS-L-FLAG but not in those expressing the two mutants (Figure 5C). However, hThrRS was readily precipitated with both wild-type and the two mutants of hThrRS-L, further confirming that its N-extension was not responsible for the interaction between hThrRS-L and hThrRS *in vivo*, consistent with data from N-extension truncated hThrRS-L (Figure 5B). We further obtained the precipitated hThrRS-L-FLAG, hThrRS-L-Ha/A-FLAG or hThrRS-L-Hb/A-FLAG as described in a previous report (30); their aminoacylation activities showed that the mutations did not affect the tRNA charging activity of hThrRS-L *in vitro* (Supplementary Figure S1). These results showed that both leucine-zippers in the N-terminal region of TLN161 (Met^1^–Cys^74^) are required to target hThrRS-L into the MSC, but have little effect on the stability and activity of hThrRS-L.

### hThrRS-L also interacts with ArgRS and the two leucine-zippers mediate interaction with ArgRS and p43

**Figure 6.**
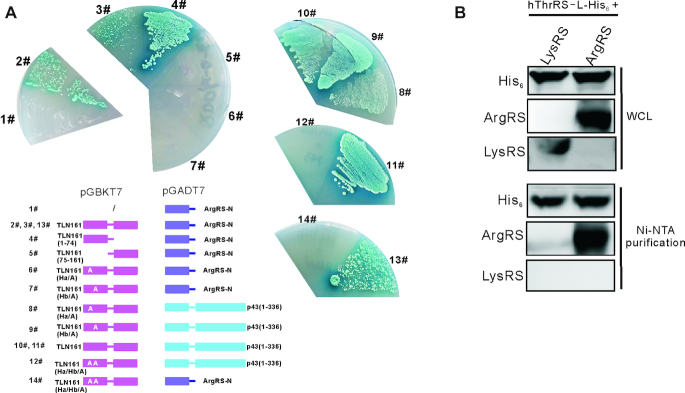
Two leucine-zippers in the N-extension mediate hThrRS-L interaction with ArgRS. (**A**) Growth of Y2H Gold yeast cells expressing either N-extension of ArgRS (ArgRS-N) alone (1#) or ArgRS-N plus TLN161 (2#, 3# and 13#) on SD/QDO/X plates. Domain mapping (4# and 5#) and leucine-zipper identification (6# and 7#) in TLN161 with ArgRS-N or with p43 (8# and 9#) were also performed using Y2H gold yeast cells by expressing various proteins or domains. In contrast to wild-type TLN161 (2#, 3# and 13# with ArgRS-N; 10# and 11# with p43), after mutation of both helixes, the resultant TLN161-Ha/Hb/A lost interaction with both p43 (12#) and ArgRS-N (14#). (**B**) Ni-NTA chromatography purification analysis confirming interaction between overexpressed ArgRS and overexpressed hThrRS-L-His_6_ in *E. coli*. As a control, LysRS and hThrRS-L-His_6_ were also overexpressed in *E. coli* and LysRS was not detected together with hThrRS-L-His_6_.

The MSC contains nine aaRSs and three auxiliary proteins. Elements responsible for MSC targeting in most aaRSs has been identified (18). To determine whether hThrRS-L interacts with other proteins in the MSC, TLN161 was used as a probe in yeast two-hybrid assays, which have successfully uncovered the interaction network in the MSC (8,18), to identify its potential interaction with most components of the MSC. To this end, we constructed eight genes encoding various proteins and peptides, comprising p18, p38, the WHEP domain of human GluProRS (Leu^753^–Asn^1023^), the N-terminal half of human cytoplasmic LysRS (Met^1^–Asp^222^), the N-terminal leucine-zipper domain of ArgRS (Met^1^–Asn^72^), the N-terminal GST domain of human MetRS (Met^1^–Ile^268^), the C-terminal appended domain of human IleRS (Asp^966^–Phe^1262^), and the C-terminal appended domain of human LeuRS (Gly^1066^–His^1176^), and then ligated them into pGADT7 separately and expressed them as fusion proteins with the GAL4 DNA activation domain in Y2HGold yeast cells expressing *TLN161*. The data showed that TLN161 clearly interacted with N-terminal domain of ArgRS (2#, 3# and 13# in Figure 6A) besides having a weak interaction with the C-terminal appended domain of LeuRS (data not shown). In addition, we observed that the N-terminal ArgRS-homologous region of TLN161 [TLN161(1–74)] (4# in Figure 6A), but not the C-terminal part of TLN161 [TLN161(75–161)] (5# in Figure 6A) was involved in the interaction between ArgRS and hThrRS-L. We further confirmed the direct interaction in *E. coli* transformants containing the genes encoding hThrRS-L-His_6_ and ArgRS after co-expression and Ni-NTA purification. Again, LysRS was not co-purified after co-expression and purification (Figure 6B).

Based on the fact that peptide Met^1^–Cys^74^ is able to interact with both ArgRS and p43, and the two leucine-zippers being responsible for hThrRS-L MSC-targeting, we further explored role of the two leucine-zippers in these interactions. When TLN161 was introduced with Ha/A or Hb/A mutations, the resultant TLN161-Ha/A or TLN161-Hb/A lost their interactions with ArgRS, suggesting that both leucine-zippers are essential for the interaction between hThrRS-L and ArgRS (6# and 7# in Figure 6A). However, both mutants interacted with p43 (8# and 9# in Figure 6A), suggesting other elements in the peptide Met^1^–Cys^74^ or either leucine-zipper is sufficient for the interaction. To test the first possibility, three TLN161 mutants in the linker region between the Ha and Hb helices were constructed [TLN161-M1/A (^34^DEQL^37^ mutated to ^34^AAAA^37^), TLN161-M2/A (^44^QAE^46^ to ^44^AAA^46^), and TLN161-M3/A (^50^LTREVAQ^56^ to ^50^AAAAAAA^56^)]. All the mutants interacted with p43 and ArgRS as well as TLN161 (data not shown). When Ha/A and Hb/A were simultaneously introduced into TLN161, the mutant TLN161-Ha/Hb/A could not interact with p43 (12# in Figure 6A) and ArgRS, as expected (14# in Figure 6A), suggesting that at least one leucine-zipper of TLN161 is necessary to interact with p43 and both leucine-zippers are crucial to interact with ArgRS.

### TLN161 is sufficient to incorporate a foreign protein into the MSC

**Figure 7.**
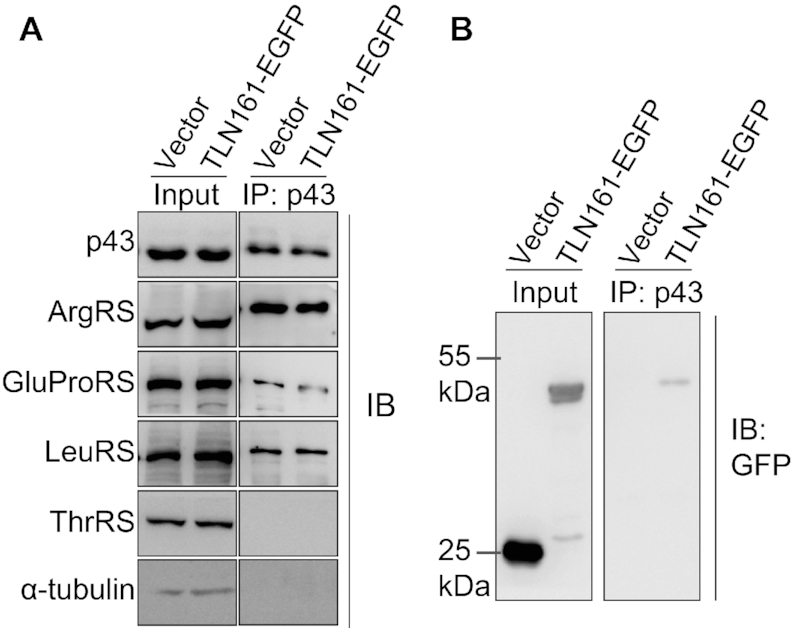
The N-extension of hThrRS-L is sufficient to assemble EGFP into MSC. EGFP or TLN161-EGFP was expressed in HEK293T cells and Co-IP was performed using p43 antibodies. Various components of MSC, hThrRS or α-Tubulin (**A**) or EGFP and TLN161-EGFP (**B**) were detected in the input or precipitated sample.

To determine whether TLN161 was sufficient to target a foreign protein into the MSC, we expressed genes encoding TLN161 fused with a C-terminal EGFP (TLN161-EGFP) or EGFP alone in HEK293T cells. The MSC was precipitated using anti-p43 antibodies, as observed by the presence of ArgRS, GluProRS and LeuRS, but the absence of ThrRS and α-tubulin in the precipitated products. The presence of TLN161-EGFP, but not EGFP, in the MSC was confirmed using anti-GFP antibodies, showing that TLN161 was sufficient to incorporate a foreign protein into the MSC (Figure 7).

### Deletion of ThrRS-L has no effect on MSC integrity *in vivo* in mouse NIH/3T3 cells

**Figure 8.**
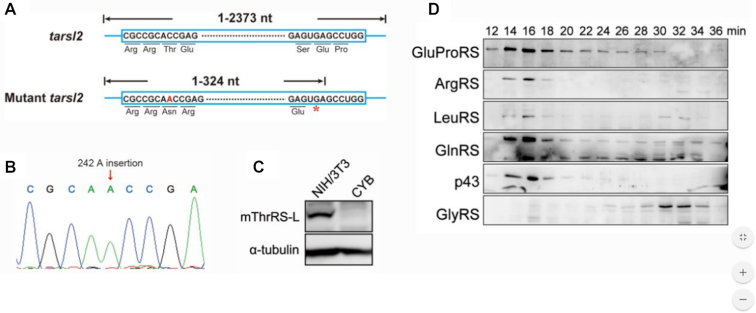
mThrRS-L does not affect MSC integrity *in vivo* in NIH/3T3. (**A**) Using CRISPR/Cas9 mediated gene editing, a *Tarsl2* deletion cell line (CYB) was obtained with an additional nucleotide ‘A’ insertion at position 242 of *Tarsl2* mRNA (**B**) The result of DNA sequencing of CYB. (**C**) mThrRS-L was not detected by western blotting because of a premature termination in translation. (**D**) Whole cell lysate of CYB was fractionated by gel filtration chromatography and various components of MSC or a free tRNA synthetase (GlyRS) were detected.

To explore any potential role of ThrRS-L in MSC integrity, especially for the assembly of p43 and ArgRS into the complex, we initially inactivated *Tarsl2* in mouse NIH/3T3 cell lines using CRISPR/Cas9. A clone (designated CYB) was obtained with a nucleotide ‘A’ insertion at position 288 of the first exon of *Tarsl2* gene (position 242 in mRNA), leading to premature termination of translation of *Tarsl2* mRNA at position 330 (Figure 8A and B). The absence of mThrRS-L was confirmed by western blotting analysis (Figure 8C).The *Tarsl2-*inactivated cell line could be obtained, which indicated that *Tarsl2* is not an essential gene in the cells. In this work, we only focused on the role of mThrRS-L in MSC assembly.

Whole cell lysate of CYB was fractionated by gel filtration chromatography to study the MSC integrity. The data clearly showed that several members of the MSC, including mouse GluProRS, LeuRS, GlnRS, and especially ArgRS and p43 were all readily incorporated into the complex; however, mouse GlyRS eluted as a free aaRS away from the MSC, as expected (Figure 8D). These data showed that the MSC remained intact at the absence of mThrRS-L, suggesting that mThrRS-L is not a pre-requisite for ArgRS and p43 incorporation into the MSC.

### Effect of potential phosphorylation at specific residues on MSC incorporation

**Figure 9.**
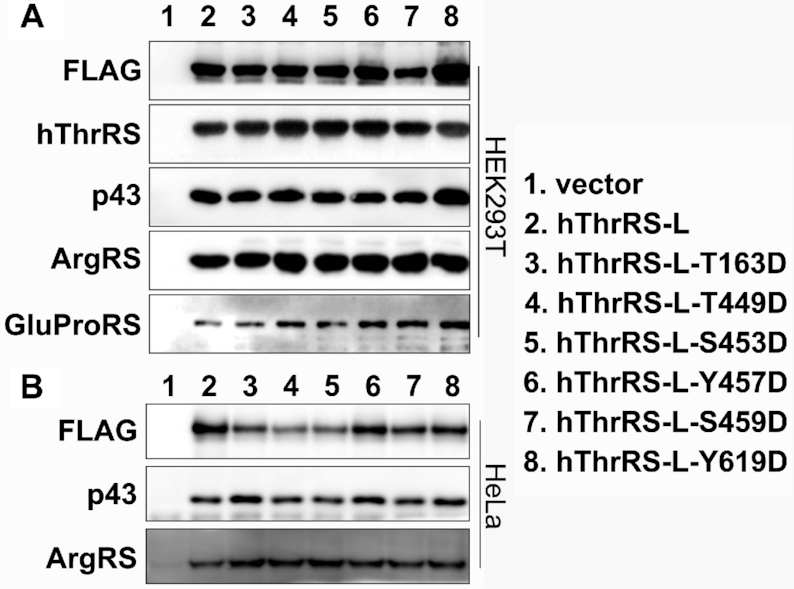
Phosphorylation of various sites of hThrRS-L has little effect on MSC targeting. Gene expressing C-terminal FLAG tagged hThrRS-L, hThrRS-L-T163D, hThrRS-L-T449D, hThrRS-L-S453D, hThrRS-L-Y457D, hThrRS-L-S459D or hThrRS-L-Y619D was separately introduced into HEK293T (**A**) or HeLa (**B**) cells. Each protein was enriched by anti-FLAG antibodies and known components of MSC and hThrRS were detected using their specific antibodies. Empty vector was also introduced as a negative control.

Post-translation modification, in particular phosphorylation, of mammalian aaRSs is frequently involved in their MSC disassociation (9,10). So far, six residues in hThrRS-L, including Thr^163^, Thr^449^, Ser^453^, Tyr^457^, Ser^459^ and Tyr^619^, has been listed to be modified by phosphorylation in the PhosphoSitePlus database (www.phosphosite.org). To address whether potential phosphorylation at these sites regulates the release of hThrRS-L from the MSC, Asp was introduced at each site to mimic phosphorylated hThrRS-L. After expressing the genes encoding these single-point mutants with a C-terminal FLAG tag in HEK29T cells, the mutants were precipitated using anti-FLAG antibodies. Several components of the MSC (including GluProRS, ArgRS and p43) were clearly detected in the precipitated products of all mutants (Figure 9A), suggesting that potential phosphorylation at each site could not disassociate hThrRS-L from the MSC. In addition, native hThrRS was also obviously present in the precipitate (Figure 9A), suggesting little effect of the potential phosphorylation of hThrRS-L on its interaction with hThrRS.

To further determine the cell specificity of incorporation of these mutants into the MSC, constructs encoding wild-type hThrRS-L and the mutants were similarly introduced into HeLa cells. In consistent with the above results, components of the MSC (ArgRS and p43) were readily detected in the precipitated products (Figure 9B). These data further showed that potential phosphorylation at each site had little effect on the targeting of hThrRS-L into the MSC.

## DISCUSSION

AaRSs from higher eukaryotes frequently acquire new appended domains at either N- or C-terminus (8). For example, hThrRS evolves a new appended domain at N-terminus when compared with bacteria ThrRSs. These newly acquired domains frequently mediate protein–protein interaction in diverse pathways, including MSC formation (8). In a previous report, we have shown that ThrRS-L gene is likely duplicated and deviated from canonical cytoplasmic ThrRS gene (30). The N-extension of human or mouse ThrRS-L was absent from ThrRSs in bacteria and lower eukaryotes and was likely of different origin from that of human or mouse cytoplasmic ThrRSs. pBlast analysis showed that the N-extension of ThrRS-L was absent from any other proteins and only displayed similarity in the first part with N-terminal extension of cytoplasmic ArgRS, in contrast to that of canonical ThrRS. Therefore, we suggest that the N-extension is a newly acquired domain during evolution.

The MSC was identified more than two decades ago (16). It is generally thought that nine aaRSs reside in the MSC, in addition to three scaffold co-factors. Indeed, these nine aaRSs have been consistently revealed as enriched in the MSC using enzymatic determination, Co-IP, silver staining, and Coomassie Brilliant Blue staining. The presence of ThrRS-L in the MSC was only noticed recently using mass spectrometry after tag purification, which is more sensitive than the above methods (28,29). In addition, we have revealed that the mRNA level of mouse *Tarsl2* is markedly lower than that of canonical mouse *Tars* in mouse tissues (ranging from 1/3 to 1/100) and cell lines (approximately 1/120) (30). Consistently, the steady-state ThrRS-L level is much lower than that of canonical aaRSs, including ThrRS and ArgRS, in HEK293T cell lines (29). The significantly low abundance of ThrRS-L relative to other aaRSs might have delayed its identification as a component of the MSC. Notably, in a recent effort to map all human protein interaction networks, 15 proteins were identified as forming a complex with ThrRS-L, four of which (p43, ArgRS, LeuRS and LysRS) are components of the MSC (39). We suggest that these four proteins were identified because of ThrRS-L’s incorporation into the MSC. This finding is also in agreement with our results showing that ThrRS-L is a *bona fide* component of the MSC. Other 11 proteins (CCDC102B, ING2, SDCBP, BATF3, RASSF7, NMI, DNA2, ALDH3B1, KIAA1683, FERMT1 and EIF4E2) are unrelated with tRNA synthetases, suggesting non-canonical functions of ThrRS-L (39).

Our data also clearly showed that the unique N-extension of ThrRS-L is responsible for its incorporation into the MSC. Two leucine-zipper motifs in the first part of the N-extension of ThrRS-L mediate its interaction with ArgRS and p43. It is notable that all leucine-zipper-containing aaRS proteins or co-factors (including ArgRS, p43 and p38) are components of the MSC and are associated in the complex, at least in part, through the leucine-zipper motifs. Therefore, our results provide a further example of leucine-zipper containing aaRS species as components of the MSC. The interaction occurs among ThrRS-L, ArgRS and p43. Indeed, after disrupting either leucine-zipper, ThrRS-L lost its ability to be incorporated into the MSC, suggesting that both leucine-zippers are essential elements for MSC localization. The N-termini of both ArgRS and p43 are long α-helices, protruding away from the main body of the proteins, as illustrated by the co-crystal structure of ArgRS-p43-GlnRS (20), making them ideal protein–protein interaction surfaces. As expected, the N-extension of ThrRS-L, especially the first half, which is similar to the N-extension of ArgRS, also likely extends out from the main body. We further modeled the structure of TLN161, which showed that the entire N-extension formed three tandem α-helices (Supplementary Figure S2). The two conserved leucine-zippers were imbedded in the first and the beginning of the second α-helix, potentially contributing to hydrophobic interactions with MSC components such as ArgRS and p43. The three termini of ArgRS, hThrRS-L, and p43 might form a highly ordered interaction network using leucine-zipper motifs in their respective N-terminal α-helices. It is expected that the shorter form of ArgRS cannot bind hThrRS-L because it lacks an N-terminal extension (35). Mutation at either leucine zipper had no effect on the protein level and enzymatic activities of ThrRS-L, suggesting that the two leucine zipper motifs have evolved for protein–protein interactions, but do not regulate the protein structure and enzymatic activities. Consistently, the activities of the shorter form of ArgRS were not decreased compared with those of the longer form of ArgRS, suggesting that the leucine zipper itself has no direct effect on enzymatic activity (40). However, the interaction between the N-terminal leucine zipper containing helices of ArgRS and p43 is important for the catalytic activity of the longer isoform (20).

Despite both ArgRS and ThrRS-L using conserved peptides to interact with p43, we believe there is little competition for the binding of p43 between ArgRS and ThrRS-L *in vivo*. This might be explained, at least in part, by the low level of ThrRS-L compared with that of ArgRS in the MSC fraction (29), making ThrRS-L a weaker competitor for ArgRS. In addition, one leucine-zipper motif can mediate an interaction with more than one partner in a single complex. For example, the leucine-zipper motif in p43 mediates its interaction with leucine zipper motifs in both ArgRS and p38 in the MSC. Furthermore, ThrRS-L and ArgRS can interact with each other using the leucine-zipper motifs in their respective N-termini. However, we have tested the leucine zipper motifs in ThrRS-L and showed that they cannot mediate its self-dimerization (data not shown). By contrast, the absence of ThrRS-L has no effect on the assembly of other components in the MSC, in line with p38 being a core scaffold of the complex (19).

Our results also explain why ThrRS, which is highly homologous to ThrRS-L over its whole sequence, is not a component of MSC: It lacks a distinct N-extension like that of ThrRS-L. However, it is striking that, despite forming a complex with ThrRS-L *in vivo*, ThrRS is not targeted to the MSC by ThrRS-L. It is possible that the interaction between ThrRS and ThrRS-L places the N-extension of ThrRS-L in an unfavorable conformation by an as-yet-unidentified mechanism (such as post-translational protein modification), stopping ThrRS-L from staying in the MSC. A similar mechanism has been observed in the assembly or release of other MSC components. For example, in monocytic cells, γ-interferon treatment induced phosphorylation at Ser886 and Ser999 of GluProRS by CDK5, disassociating it from the MSC to bind other protein partners and forming a GAIT complex to suppress the translation of various inflammatory mRNAs (9). Upon laminin signaling, LysRS is phosphorylated at Thr52 by p38MAPK, which disassociates it from the MSC to the cell membrane to interact with 67LR, thereby regulating cell migration (41). LysRS binds with MITF to regulate the immune response in mast cells after it is released from the MSC upon phosphorylation at Ser207 (42). In addition, our previous report showed that some fraction of ThrRS-L is localized in the nucleus (30). We suggest that under most conditions, ThrRS-L is incorporated in the MSC; but under some specific but unidentified conditions, ThrRS-L is modified and then translocated to the nucleus; the nucleus-localized ThrRS-L is likely not associated with the MSC.

Our recent data from native or overexpressed ThrRS-L showed that ThrRS-L could catalyze both aminoacylation and editing functions *in vitro* (30). However, we hypothesized that ThrRS-L has not evolved for canonical tRNA aminoacylation for protein synthesis because of its presence only in higher eukaryotes, the presence of a conserved *TARS* in the three domains of life, and the lower abundance of ThrRS-L *in vivo* (29,30). We also suggested that it is unlikely to solely catalyze editing activity to hydrolyze mischarged Ser-tRNA^Thr^*in vivo* under physiological conditions because, on the one hand, ThrRS itself retains the editing activity and on the other hand, it would not be evolutionarily economical to evolve a new gene just to perform another layer of editing. However, we cannot rule out the possibility that its residence in the MSC might be beneficial for recycling tRNA^Thr^ to supplement ThrRS activity to some extent under specific conditions (such as under stress or when ThrRS is secreted outside of cells during angiogenesis) (43). In addition, its presence in the nucleus and its potential to bind both nucleic acid (via the tRNA binding domain) and proteins (via the leucine-zipper motifs) strongly implied that it has some non-canonical functions, at least in the nucleus, which are currently under investigation. The MSC might function as a reservoir to regulate its cellular localization, similar to LysRS and GluProRS, as one of its non-canonical functions (22).

## Supplementary Material

gkz588_Supplemental_File

## References

[1] Giege R., Springer M. Aminoacyl-tRNA synthetases in the bacterial world. EcoSal Plus. 2016; 7:doi:10.1128/ecosalplus.ESP-0002-2016. 10.1128/ecosalplus.esp-0002-2016 PMC11575706 27223819

[2] Ibba M., Soll D. Aminoacyl-tRNA synthesis. Annu. Rev. Biochem. 2000; 69:617–650. 10966471 10.1146/annurev.biochem.69.1.617

[3] Guo M., Yang X.L., Schimmel P. New functions of aminoacyl-tRNA synthetases beyond translation. Nat. Rev. Mol. Cell Biol. 2010; 11:668–674. 20700144 10.1038/nrm2956 PMC3042954

[4] Zhou X., Wang E. Transfer RNA: a dancer between charging and mis-charging for protein biosynthesis. Sci. China Life Sci. 2013; 56:921–932. 23982864 10.1007/s11427-013-4542-9

[5] Ling J., Reynolds N., Ibba M. Aminoacyl-tRNA synthesis and translational quality control. Annu. Rev. Microbiol. 2009; 63:61–78. 19379069 10.1146/annurev.micro.091208.073210

[6] Eriani G., Delarue M., Poch O., Gangloff J., Moras D. Partition of tRNA synthetases into two classes based on mutually exclusive sets of sequence motifs. Nature. 1990; 347:203–206. 2203971 10.1038/347203a0

[7] Cusack S. Aminoacyl-tRNA synthetases. Curr. Opin. Struct. Biol. 1997; 7:881–889. 9434910 10.1016/s0959-440x(97)80161-3

[8] Guo M., Schimmel P., Yang X.L. Functional expansion of human tRNA synthetases achieved by structural inventions. FEBS Lett. 2010; 584:434–442. 19932696 10.1016/j.febslet.2009.11.064 PMC2826164

[9] Arif A., Jia J., Mukhopadhyay R., Willard B., Kinter M., Fox P.L. Two-site phosphorylation of EPRS coordinates multimodal regulation of noncanonical translational control activity. Mol. Cell. 2009; 35:164–180. 19647514 10.1016/j.molcel.2009.05.028 PMC2752289

[10] Ofir-Birin Y., Fang P., Bennett S.P., Zhang H.M., Wang J., Rachmin I., Shapiro R., Song J., Dagan A., Pozo J. et al. Structural switch of lysyl-tRNA synthetase between translation and transcription. Mol. Cell. 2013; 49:30–42. 23159739 10.1016/j.molcel.2012.10.010 PMC3766370

[11] Wakasugi K., Schimmel P. Two distinct cytokines released from a human aminoacyl-tRNA synthetase. Science. 1999; 284:147–151. 10102815 10.1126/science.284.5411.147

[12] Kim S., You S., Hwang D. Aminoacyl-tRNA synthetases and tumorigenesis: more than housekeeping. Nat. Rev. Cancer. 2011; 11:708–718. 21941282 10.1038/nrc3124

[13] Park S.G., Schimmel P., Kim S. Aminoacyl tRNA synthetases and their connections to disease. Proc. Natl. Acad. Sci. U.S.A. 2008; 105:11043–11049. 18682559 10.1073/pnas.0802862105 PMC2516211

[14] Galani K., Grosshans H., Deinert K., Hurt E.C., Simos G. The intracellular location of two aminoacyl-tRNA synthetases depends on complex formation with Arc1p. EMBO J. 2001; 20:6889–6898. 11726524 10.1093/emboj/20.23.6889 PMC125769

[15] Praetorius-Ibba M., Hausmann C.D., Paras M., Rogers T.E., Ibba M. Functional association between three archaeal aminoacyl-tRNA synthetases. J. Biol. Chem. 2007; 282:3680–3687. 17158871 10.1074/jbc.M609988200

[16] Kerjan P., Cerini C., Semeriva M., Mirande M. The multienzyme complex containing nine aminoacyl-tRNA synthetases is ubiquitous from Drosophila to mammals. Biochim. Biophys. Acta. 1994; 1199:293–297. 8161568 10.1016/0304-4165(94)90009-4

[17] Filonenko V.V., Deutscher M.P. Evidence for similar structural organization of the multienzyme aminoacyl-tRNA synthetase complex in vivo and in vitro. J. Biol. Chem. 1994; 269:17375–17378. 8021235

[18] Rho S.B., Kim M.J., Lee J.S., Seol W., Motegi H., Kim S., Shiba K. Genetic dissection of protein–protein interactions in multi-tRNA synthetase complex. Proc. Natl. Acad. Sci. U.S.A. 1999; 96:4488–4493. 10200289 10.1073/pnas.96.8.4488 PMC16359

[19] Kim J.Y., Kang Y.S., Lee J.W., Kim H.J., Ahn Y.H., Park H., Ko Y.G., Kim S. p38 is essential for the assembly and stability of macromolecular tRNA synthetase complex: implications for its physiological significance. Proc. Natl. Acad. Sci. U.S.A. 2002; 99:7912–7916. 12060739 10.1073/pnas.122110199 PMC122994

[20] Fu Y., Kim Y., Jin K.S., Kim H.S., Kim J.H., Wang D., Park M., Jo C.H., Kwon N.H., Kim D. et al. Structure of the ArgRS-GlnRS-AIMP1 complex and its implications for mammalian translation. Proc. Natl. Acad. Sci. U.S.A. 2014; 111:15084–15089. 25288775 10.1073/pnas.1408836111 PMC4210331

[21] Sivaram P., Deutscher M.P. Existence of two forms of rat liver arginyl-tRNA synthetase suggests channeling of aminoacyl-tRNA for protein synthesis. Proc. Natl. Acad. Sci. U.S.A. 1990; 87:3665–3669. 2187187 10.1073/pnas.87.10.3665 PMC53963

[22] Ray P.S., Arif A., Fox P.L. Macromolecular complexes as depots for releasable regulatory proteins. Trends Biochem. Sci. 2007; 32:158–164. 17321138 10.1016/j.tibs.2007.02.003

[23] Han J.M., Jeong S.J., Park M.C., Kim G., Kwon N.H., Kim H.K., Ha S.H., Ryu S.H., Kim S. Leucyl-tRNA synthetase is an intracellular leucine sensor for the mTORC1-signaling pathway. Cell. 2012; 149:410–424. 22424946 10.1016/j.cell.2012.02.044

[24] Ko Y.G., Kim E.Y., Kim T., Park H., Park H.S., Choi E.J., Kim S. Glutamine-dependent antiapoptotic interaction of human glutaminyl-tRNA synthetase with apoptosis signal-regulating kinase 1. J. Biol. Chem. 2001; 276:6030–6036. 11096076 10.1074/jbc.M006189200

[25] Ko Y.G., Kang Y.S., Kim E.K., Park S.G., Kim S. Nucleolar localization of human methionyl-tRNA synthetase and its role in ribosomal RNA synthesis. J. Cell Biol. 2000; 149:567–574. 10791971 10.1083/jcb.149.3.567 PMC2174846

[26] Antonellis A., Green E.D. The role of aminoacyl-tRNA synthetases in genetic diseases. Annu. Rev. Genomics Hum. Genet. 2008; 9:87–107. 18767960 10.1146/annurev.genom.9.081307.164204

[27] Zhou X.L., Ruan Z.R., Huang Q., Tan M., Wang E.D. Translational fidelity maintenance preventing Ser mis-incorporation at Thr codon in protein from eukaryote. Nucleic Acids Res. 2013; 41:302–314. 23093606 10.1093/nar/gks982 PMC3592468

[28] Kim K., Park S.J., Na S., Kim J.S., Choi H., Kim Y.K., Paek E., Lee C. Reinvestigation of aminoacyl-tRNA synthetase core complex by affinity purification-mass spectrometry reveals TARSL2 as a potential member of the complex. PLoS One. 2013; 8:e81734. 24312579 10.1371/journal.pone.0081734 PMC3846882

[29] Park S.J., Ahn H.S., Kim J.S., Lee C. Evaluation of multi-tRNA Synthetase complex by multiple reaction monitoring mass spectrometry coupled with size exclusion chromatography. PLoS One. 2015; 10:e0142253. 26544075 10.1371/journal.pone.0142253 PMC4636271

[30] Chen Y., Ruan Z.R., Wang Y., Huang Q., Xue M.Q., Zhou X.L., Wang E.D. A threonyl-tRNA synthetase-like protein has tRNA aminoacylation and editing activities. Nucleic Acids Res. 2018; 46:3643–3656. 29579307 10.1093/nar/gky211 PMC5909460

[31] Lei H.Y., Zhou X.L., Ruan Z.R., Sun W.C., Eriani G., Wang E.D. Calpain cleaves most components in the multiple aminoacyl-tRNA synthetase complex and affects their functions. J. Biol. Chem. 2015; 290:26314–26327. 26324710 10.1074/jbc.M115.681999 PMC4646279

[32] Ran F.A., Hsu P.D., Wright J., Agarwala V., Scott D.A., Zhang F. Genome engineering using the CRISPR-Cas9 system. Nat. Protoc. 2013; 8:2281–2308. 24157548 10.1038/nprot.2013.143 PMC3969860

[33] Zhou X.L., Ruan Z.R., Wang M., Fang Z.P., Wang Y., Chen Y., Liu R.J., Eriani G., Wang E.D. A minimalist mitochondrial threonyl-tRNA synthetase exhibits tRNA-isoacceptor specificity during proofreading. Nucleic Acids Res. 2014; 42:13873–13886. 25414329 10.1093/nar/gku1218 PMC4267643

[34] Zhou X.L., Chen Y., Fang Z.P., Ruan Z.R., Wang Y., Liu R.J., Xue M.Q., Wang E.D. Translational quality control by bacterial threonyl-tRNA synthetases. J. Biol. Chem. 2016; 291:21208–21221. 27542414 10.1074/jbc.M116.740472 PMC5076528

[35] Zheng Y.G., Wei H., Ling C., Xu M.G., Wang E.D. Two forms of human cytoplasmic arginyl-tRNA synthetase produced from two translation initiations by a single mRNA. Biochemistry. 2006; 45:1338–1344. 16430231 10.1021/bi051675n

[36] Ruan Z.R., Fang Z.P., Ye Q., Lei H.Y., Eriani G., Zhou X.L., Wang E.D. Identification of lethal mutations in yeast threonyl-tRNA synthetase revealing critical residues in its human homolog. J. Biol. Chem. 2015; 290:1664–1678. 25416776 10.1074/jbc.M114.599886 PMC4340410

[37] Park S.G., Choi E.C., Kim S. Aminoacyl-tRNA synthetase-interacting multifunctional proteins (AIMPs): a triad for cellular homeostasis. IUBMB Life. 2010; 62:296–302. 20306515 10.1002/iub.324

[38] Shalak V., Kaminska M., Mirande M. Translation initiation from two in-frame AUGs generates mitochondrial and cytoplasmic forms of the p43 component of the multisynthetase complex. Biochemistry. 2009; 48:9959–9968. 19775078 10.1021/bi901236g

[39] Huttlin E.L., Bruckner R.J., Paulo J.A., Cannon J.R., Ting L., Baltier K., Colby G., Gebreab F., Gygi M.P., Parzen H. et al. Architecture of the human interactome defines protein communities and disease networks. Nature. 2017; 545:505–509. 28514442 10.1038/nature22366 PMC5531611

[40] Kyriacou S.V., Deutscher M.P. An important role for the multienzyme aminoacyl-tRNA synthetase complex in mammalian translation and cell growth. Mol. Cell. 2008; 29:419–427. 18313381 10.1016/j.molcel.2007.11.038 PMC2273998

[41] Kim D.G., Choi J.W., Lee J.Y., Kim H., Oh Y.S., Lee J.W., Tak Y.K., Song J.M., Razin E., Yun S.H. et al. Interaction of two translational components, lysyl-tRNA synthetase and p40/37LRP, in plasma membrane promotes laminin-dependent cell migration. FASEB J. 2012; 26:4142–4159. 22751010 10.1096/fj.12-207639

[42] Yannay-Cohen N., Carmi-Levy I., Kay G., Yang C.M., Han J.M., Kemeny D.M., Kim S., Nechushtan H., Razin E. LysRS serves as a key signaling molecule in the immune response by regulating gene expression. Mol. Cell. 2009; 34:603–611. 19524539 10.1016/j.molcel.2009.05.019

[43] Williams T.F., Mirando A.C., Wilkinson B., Francklyn C.S., Lounsbury K.M. Secreted threonyl-tRNA synthetase stimulates endothelial cell migration and angiogenesis. Sci. Rep. 2013; 3:1317. 23425968 10.1038/srep01317 PMC3578223

