# Supplementary material for: Newly acquired N-terminal extension targets threonyl-tRNA synthetase-like protein into the multiple tRNA synthetase complex
Source: Nucleic Acids Res. 2019 Jul 9;47(16):8662–74. doi: 10.1093/nar/gkz588 (PMC6794377; doi:10.1093/nar/gkz588)
Supplement: gkz588_Supplemental_File [file gkz588_supplemental_file.docx]

**
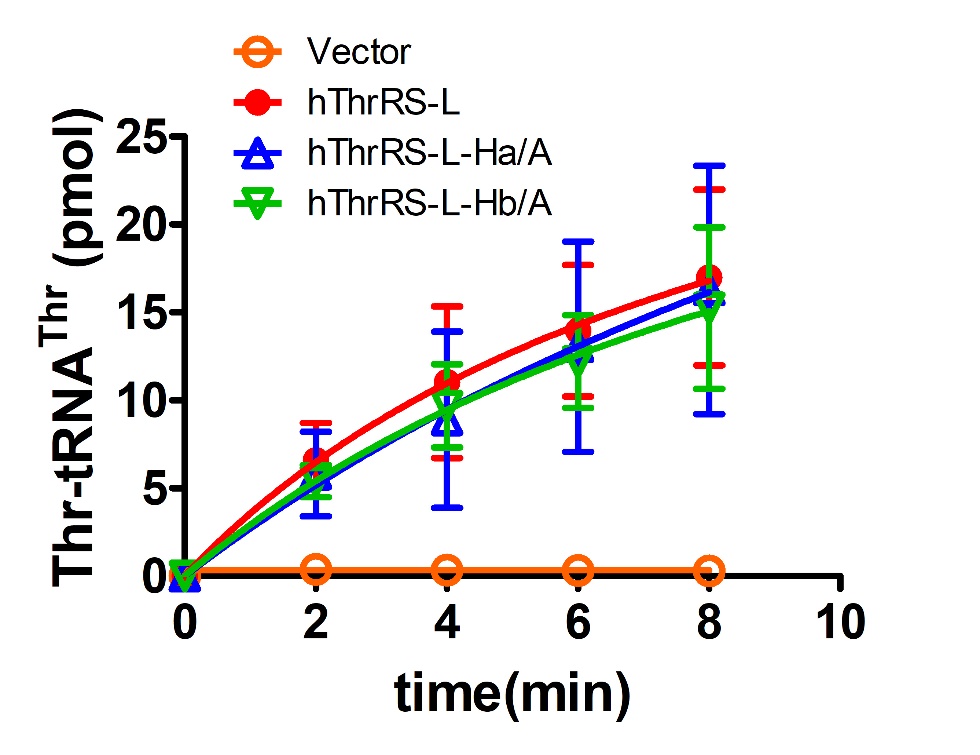
**

**Supplementary Figure 1. Aminoacylation activities of hThrRS-L and two leucine-zipper mutants.**

FLAG-tagged hThrRS-L, hThrRS-L-Ha/A, and hThrRS-L-Hb/A were enriched from their overexpressing HEK293T cells and their aminoacylation activities were determined with the packed precipitated beads. Data represent averages with SD indicated.


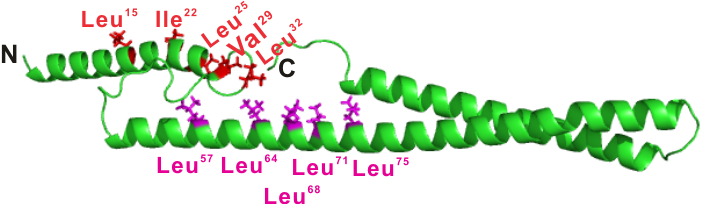


**Supplementary Figure 2. Model of TNL161.**

The structure model of residues 1-161 of hThrRS-L was predicted with the program I-TASSER, and geometry-minimized with the software PHENIX. Side chains of residues in the two leucine-zippers were shown and colored red and pink, respectively. N, N-terminus; C, C-terminus.
